# Increased Lead Biomarker Levels Are Associated with Changes in Hormonal Response to Stress in Occupationally Exposed Male Participants

**DOI:** 10.1289/ehp.1103873

**Published:** 2011-11-23

**Authors:** Marie C. Fortin, Deborah A. Cory-Slechta, Pamela Ohman-Strickland, Chizoba Nwankwo, T. Steven Yanger, Andrew C. Todd, Jan Moynihan, James Walton, Andrew Brooks, Nancy Fiedler

**Affiliations:** 1 Environmental and Occupational Health Sciences Institute, Robert Wood Johnson Medical School, University of Medicine and Dentistry of New Jersey, Piscataway, New Jersey, USA; 2 Department of Environmental Medicine, University of Rochester School of Medicine, Rochester, New York, USA; 3 Environmental and Occupational Health Sciences Institute, School of Public Health, University of Medicine and Dentistry of New Jersey, Piscataway, New Jersey, USA; 4 Department of Epidemiology, School of Public Health, University of Medicine and Dentistry of New Jersey, Piscataway, New Jersey, USA; 5 Department of Preventive Medicine, Mount Sinai School of Medicine, New York, New York, USA; 6 Department of Psychiatry, University of Rochester School of Medicine, Rochester, New York, USA; 7 Department of Genetics, Rutgers, The State University of New Jersey, Piscataway, New Jersey, USA

**Keywords:** ACTH, cortisol, lead, Pb, Trier Social Stress Test

## Abstract

Background: Lead (Pb) exposure has been associated with a host of pathological conditions in humans. In rodents Pb exposure has been shown to alter the hypothalamic–pituitary–adrenal (HPA) axis function.

Objective: We investigated the effects of lead on responses of the HPA axis to a psychosocial laboratory stressor administered to Pb-exposed workers.

Methods: Seventy male participants completed the Trier Social Stress Test (TSST). Serum cortisol (CORT) and plasma adrenocorticotropic hormone (ACTH) were assessed in response to and during recovery from the stressor. We measured Pb in blood, a biomarker of recent exposure, and in tibia bone by X-ray fluorescence (XRF), a biomarker of chronic exposure.

Results: The TSST induced statistically significant increases in ACTH and CORT in the participants. At baseline, ACTH was not significantly higher (*p* = 0.052) in participants with higher blood Pb concentration, but CORT was significantly lower in these participants (*p* = 0.016). Adjusted linear regression models indicated a positive association between blood and bone Pb and the increase in ACTH in response to stress. However, Pb was not strongly associated with changes in CORT in response to stress. Pb was also associated with the ACTH:CORT ratio at baseline and throughout the course of the protocol, suggesting an adrenal hyporesponsiveness in participants with higher Pb concentrations.

Conclusion: The altered HPA-axis stress response observed in participants exposed to higher levels of Pb further supports the idea that lead may contribute to a host of biological dysfunctions beyond the classical neurotoxic effects.

Although blood lead (BPb) levels have dramatically decreased in the general population (Annest et al. 1983; Centers for Disease Control and Prevention 2009), lead (Pb) exposure remains an issue in specific population clusters, such as low socioeconomic inner-city neighborhoods (Haley and Talbot 2004), and in occupationally exposed workers. Pb is a well-known neurotoxicant (Shih et al. 2007; van Wijngaarden et al. 2009) but has also been linked to hypertension in humans (Navas-Acien et al. 2007; Vaziri and Gonick 2008; Weaver et al. 2008) and to alterations in the hypothalamic–pituitary–adrenal (HPA) axis in animals.

The HPA axis is critical to homeostatic function and plays an essential role in mediating the body’s stress response. HPA-axis dysfunction has been associated with multiple disease states, including depression (Carroll et al. 2007), metabolic disease and obesity (Reynolds 2010), and hypertension (Wirtz et al. 2007). Both hypo- and hyperfunction of the HPA axis alter metabolic and cardiovascular function. In humans, the major circulating components of the HPA axis are *a*) adrenocorticotropic hormone (ACTH), which is released into the bloodstream by the pituitary gland in response to the release of corticotropin-releasing hormone (CRH) by the hypothalamus, and *b*) cortisol (CORT), which is released into blood by the adrenal glands following stimulation by ACTH. ACTH and CORT can thus be used as biomarkers of HPA axis function.

In animals, initial elevated corticosterone concentrations were observed in rats developmentally exposed to Pb (Virgolini et al. 2008), and a significant delay in the glucocorticoid negative feedback has been reported in developmentally exposed rodents (Rossi-George et al. 2009). Male offspring of rat dams exposed to low-level Pb were reported to have prolonged elevation of corticosterone levels following a dexamethasone challenge test (Rossi-George et al. 2009). These HPA axis alterations were modulated by sex and prenatal stress.

Such findings underscore the importance of determining whether, and under what conditions, these effects translate to human populations. Recently, Gump et al. (2008) examined whether low-level Pb exposure [measured in cord blood at birth (median, 1.4 µg/dL) and in blood at 2.6 years of age (median, 4.1 µg/dL)] would alter salivary cortisol responses to an acute stress challenge in children at 9.5 years of age. After statistical adjustments for designated covariates, higher cord Pb and BPb levels were associated with enhanced salivary cortisol response to the stress challenge—effects consistent with the delay in glucocorticoid negative feedback seen in rats.

The purpose of the present study was to investigate HPA axis function among adult workers with a history of occupational exposure to Pb. Specifically, we examined ACTH and CORT at rest and in response to the Trier Social Stress Test (TSST), a psychosocial laboratory stressor.

## Methods

*Subject recruitment.* This study was approved by the Institutional Review Board of the University of Medicine and Dentistry of New Jersey (UMDNJ)–Robert Wood Johnson Medical School. Information about the project was distributed at union meetings and through mailings to union members from the International Union of Painters and Allied Trades (District Council 9, New York, NY) and to dry wall/tapers and glaziers (District Council 21, Philadelphia, PA) and carpenters (New Jersey). Recruiting within these groups ensured a range of Pb exposures, with carpenters being typically less exposed than painters. Individuals who contacted our office were screened with a standardized telephone interview, and those who reported any of the following conditions were not recruited to participate: neurologic disease or brain injury, stroke or cardiovascular disease (including diagnosed hypertension), serious pulmonary disease (including asthma), liver or kidney disease, serious gastrointestinal disorders, known endocrine disease, and major psychiatric conditions (psychoses, bipolar disorder, alcoholism, or drug abuse). In addition, individuals taking psychoactive drugs, beta-blockers, or glucocorticoids were not recruited to participate. Participants were scheduled for two separate appointments to complete the experimental stress procedure and a bone lead measurement. Of the 134 individuals who contacted our offices, 39 were not interested in participating, lived out-of-state, or failed to come to their scheduled appointment. In addition, 19 were ineligible because of diagnosed cardiovascular disease (*n* = 12), severe asthma (*n* = 2), diabetes (*n* = 1), or < 10 years of experience in their trade (*n* = 4). Finally, 76 individuals were scheduled for the stress protocol.

*Biological measures, questionnaires and stress challenge.* BPb and bone Pb measurements. BPb was assessed by Quest Diagnostics (Teterboro, NJ) as a part of routine blood chemistries. Tibia lead (TPb), which was used as an indicator of cumulative (chronic) Pb exposure, was measured by ^109^Cd-based K-shell X-ray fluorescence (XRF) at the Mount Sinai Medical Center by A.C.T., as described previously (Schwartz et al. 1999; Todd et al. 1993). Thirty-minute measurements were taken at the midshaft of the left tibia after the region was washed with a 3% solution of dilute glacial acetic acid. Participants were asked to sit still while measurements were taken. BPb and TPb measurements are expressed as micrograms of Pb per deciliter of blood and micrograms of Pb per gram of bone mineral, respectively. We collected additional blood samples to perform genotyping for polymorphisms relevant to the HPA axis and Pb biodisposition (results not shown).

ACTH and cortisol. We collected blood (3–5 mL) in tubes containing no additives for assaying CORT, and in tubes containing EDTA for assaying ACTH. We placed the tubes on ice, centrifuged them at 3,000 rpm for 15 min, and stored 500-µL aliquots of serum or plasma at –80°C until assayed. We measured serum CORT and plasma ACTH with competitive enzyme immunoassay kits (Cortisol ELISA and ACTH ELISA; ALPCO Diagnostics, Salem, NH) following the manufacturer’s protocols. The CORT assay is sensitive to 0.4 µg/dL and the ACTH assay to 0.46 pg/mL. The intra- and interassay coefficients of variation reported by the manufacturer ranged between 2.3 and 9.4%.

Questionnaires. A cumulative lifetime solvent exposure index (SEI) was estimated based on work history, application methods, and use of protective equipment for each subject who reported ever working with solvent-based paints, as described in detail by Wang et al. (2011). For current smokers, lifetime nicotine use was calculated in pack-years of smoking. Current and past alcohol and drug use (e.g. marijuana, stimulants, cocaine) was estimated as described previously (Fiedler et al. 2003). We used the DS14 to assess negative affect and social inhibition (components of type D personality; DeFruyt and Denollet 2002); the Psychiatric Epidemiology Research Interview (PERI) to assess stressful life events and chronic stress; and the Daily Hassles Scale to assess the frequency of daily problems and the average intensity of distress associated with these hassles (DeLongis et al. 1982; Kanner et al. 1981). Finally, we assessed state anxiety using the State-Trait Anxiety Inventory (STAI; Spielberger et al. 1983) and depression using the Beck Depression Inventory (BDI-II; Beck 1996).

Stress challenge. The TSST (Kirschbaum et al. 1993) consists of *a*) a 5-min speech during which the participant introduces himself and explains why he is the ideal candidate for an hypothetical job position, and *b*) a 5-min mental arithmetic task (serial subtraction of 13 from 1,022). In the 5 min preceding the speech, participants are given the instructions and told to use the remaining time to prepare. According to the standardized protocol, participants were observed by an audience of one examiner and two observers and videotaped to increase the level of stress. Participants were also told that staff members of the laboratory would later evaluate performance by analyzing the videotapes. In the event a subject finished his speech in less than the required 5 min, the examiner responded in a standardized manner by telling the subject the following: “You still have some time left; please continue.” When a subject made a mistake during the serial subtraction, the examiner told him to stop and start again from the initial number.

*Procedure.* We tested the participants in the afternoon [participants arrived at the research facilities at about 1120 hours ± 1 hr and the protocol (catheterization) began at about 1225 hours ± 1 hr] to maximize stress responsivity (Dickerson and Kemeny 2004) and to limit the effects of circadian rhythm. Participants were asked not to exercise or use alcohol or over-the-counter medications for 24 hr, not to use caffeine for 12 hr, and not to smoke for 2 hr, which they confirmed upon admission. Acutely ill participants were rescheduled. Upon arrival, we greeted the participants and explained the study, reviewed the consent forms with them, and answered all their questions. Individuals willing to participate gave written informed consent.

**Figure 1:**
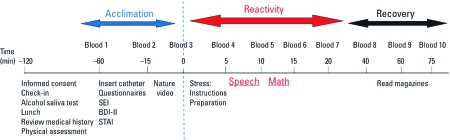
Protocol timeline. Blood was sampled 10 times during the protocol. Other questionnaires were self-administered at home before the laboratory session.

The experimental sequence is provided in Figure 1. Briefly, the research nurse ascertained that the participant met the inclusion criteria and administered an alcohol saliva test (Q.E.D. Saliva Alcohol Test A150; OraSure Technologies Inc. Bethlehem, PA) to rule out acute effects of alcohol. Then, we gave participants a light lunch and reviewed the questionnaires they had completed at home to ensure accuracy. We measured each participant’s height, weight, and blood pressure and placed a catheter in the antecubital vein of the participant’s nondominant arm, which marked time –60 min. Blood was then drawn for the standard chemistry panel and BPb. From –60 min to 0 min, the participant acclimated to the experimental setting, completed the other questionnaires, and watched a nature video. At time 0 we collected the “baseline” samples and then gave the instructions for the TSST; this initiated the stress reactivity period. From approximately minute 20 to minute 75, the participants recovered while reading preselected magazines. Blood samples were collected during the acclimation, stress reactivity, and recovery periods. After the last blood collection, the participants were debriefed to explain that their performance “was not” and “will not be” evaluated and that the only purpose of the TSST was to initiate a stress reaction.

*Statistical analysis.* We summarized the distributions of all variables (predictors and outcomes) using means, SEs, percentiles, and histograms. CORT, ACTH, BPb, and TPb were right-skewed and log-transformed (adding one to variables having at least one zero observation) for the purposes of inference.

We used paired *t*-tests to contrast log-ACTH and log-CORT levels before and after the TSST. We used both continuous linear regression and analysis of covariance to estimate effects of log-BPb and log-TPb on log-ACTH and log-CORT measurements and on the log-ratio of ACTH:CORT at each time point. In addition, we estimated associations between Pb and log-transformed, time-weighted average values of ACTH and CORT over all time points [i.e., the area under the curve (AUC)] using regression models.

Models of outcomes measured after time 0 were run with and without adjustment for their respective baseline level. Adjustment for baseline allowed us to look specifically at stress reactivity. We used the regression parameters to estimate the percent increase or decrease in response (ACTH, CORT) associated with an interquartile range (IQR) increase in log-transformed Pb (with log-BPb or log-TPb modeled as continuous variables) and associated with Pb being above versus below the median value (i.e., with BPb or TPb modeled as dichotomous variables).

Five participants who failed to show ACTH and CORT response to the TSST were defined as nonresponders. More specifically, these participants’ ACTH and CORT levels did not rise above their own baseline following the administration of the TSST instructions or at any time thereafter (values were equal or lower than baseline). We conducted a parallel set of statistical analyses excluding nonresponders to determine whether including these men influenced the observed associations.

## Results

*Participants.* Among the 76 scheduled participants, 6 were not included in the statistical analysis: 1 had a positive alcohol saliva test; consistent blood draw could not be achieved for 3 individuals; and 2 were females. The females were excluded because of the potential sex differences that could not be addressed due to the very small sample size. Thus, 70 participants (46 construction painters; 24 carpenters/dry-wall/tapers) completed the protocol; 49 also completed the TPb measurements.

*Biomarkers of lead exposure.* In the present study, BPb levels ranged from below the limit of detection (LOD) of 3 µg/dL of blood to 31 µg/dL. The 25th, 50th, and 75th percentiles of BPb distribution were 3 µg/dL, 4 µg/dL and 7 µg/dL, respectively. TPb levels ranged between below the LOD of 5 µg/g to 70 µg/g of bone. The 25th, 50th, and 75th percentiles of TPb distribution were 11 µg/g, 13 µg/g, and 17 µg/g respectively. The correlation between BPb and TPb was 0.148 (Spearman rho; *p* = 0.316).

*Demographic variables and covariates.* We divided participants into high and low BPb and TPb groups based on a median split and compared characteristics between these groups (Table 1). Participants with higher BPb levels had less education and higher levels of depression, negative affect, social inhibition, and SEI (*p* < 0.05). ACTH, CORT, ACTH:CORT ratio, BPb level, and TPb level were log-transformed for the statistical analyses. Age, education, negative affect, social inhibition (DS14), and depression (BDI-II) were associated with BPb levels (*p* < 0.1) and were included as covariates in the adjusted models. SEI was also associated with BPb, but it was not included in the final adjusted models because exposure to solvents could be viewed as an indirect measure of exposure to lead (the two exposures are co-occurring) and is also associated with age.

**Table 1:** Participant characteristics for low and high BPb*^a^* groups and for the two groups combined.

Combined					Low BPb					High BPb				
Variable	*n*	Mean (95% CI)	Range	*n*	Mean (95% CI)	Range	*n*	Mean (95% CI)	Range	*p*-Value*b*
Age (years)		70		46.4 (44.8, 48.1)		30–60		36		47.8 (45.5, 50.2)		32–59		34		45.0 (42.6, 47.4)		30–60		0.090
Years worked		70		20.2 (18.5, 21.9)		9–35		36		21.5 (19.1, 23.9)		10–35		34		18.8 (16.3, 21.3)		9–34		0.11
Years of education		69		12.6 (12.3, 12.9)		9–16		35		13.0 (12.6, 13.4)		11–16		34		12.1 (11.7, 12.6)		9–15		0.0028
BMI		70		28.7 (27.8, 29.7)		18.5–36.5		36		28.5 (27.1, 30.3)		18.5–36.5		34		28.8 (27.7, 29.9)		21.9–35.0		0.80
Smoking pack-years (among smokers)		40		20.6 (15.3, 25.9)		1.4–77.5		18		24.3 (14.4, 34.2)		1.4–77.5		22		17.6 (11.9, 23.3)		1.5–49.5		0.62
Lifetime use																				
Alcohol (no. of drinks)		70		22,789 (13,175, 32,403)		0–186,880		36		24,571 (91,50, 39,993)		0–186,880		34		20,903 (8,872, 32,933)		0–141,733		0.70
Marijuana (times used)		70		549 (209, 889)		0–8,030		36		614 (165, 1062)		0–5,110		34		480 (0, 1,019)		0–8,030		0.70
Cocaine (times used)		70		152 (0, 324)		0–5,222		36		108 (0, 280)		0–2,958		34		198 (0, 515)		0–5,222		0.61
Scale (score)																				
Depression (BDI-II)*c*		70		5.5 (4.2, 6.8)		0–32		36		4.1 (2.9, 5.3)		0–11.0		34		6.9 (4.6, 9.2)		0–32		0.034
Negative affect*d*		70		6.0 (4.8, 7.1)		0–19		36		4.0 (2.8, 5.2)		0–13.0		34		8.1 (6.2, 9.9)		0–19		0.0004
Social inhibition*e*		70		8.7 (7.3, 10.2)		0–22		36		6.6 (5.1, 8.2)		0–16.0		34		10.9 (8.6, 13.2)		0–22		0.0026
State anxiety*f*		70		31.1 (20.9, 33.3)		20–51		36		30.6 (27.6, 33.6)		20–50		34		31.5 (28.2, 44.8)		20–51		0.69
Daily hassles																				
Frequency		63		21.4 (16.1, 26.7)		0–115		30		17.1 (12.1, 22.2)		0–59		33		25.3 (16.2, 34.4)		0–115		0.12
Mean intensity		63		1.5 (1.4, 1.6)		0–2.9		30		1.5 (1.3, 1.8)		0–2.9		33		1.5 (1.3, 1.6)		0–2.3		0.60
Life events																				
Frequency		63		2.4 (1.6, 3.3)		0–15		32		2.3 (1.2, 3.4)		0–15		31		2.6 (1.4, 3.9)		0–12		0.69
Mean severity		63		1.8 (1.4, 2.3)		0–5		32		1.8 (1.3, 2.4)		0–5		31		1.9 (1.2, 2.5)		0–5		0.97
SEI		70		3.7 (1.9, 5.5)		0–33.4		36		1.5 (0.4, 2.6)		0–12.9		34		6.0 (2.5, 9.4)		0–33.4		0.016
Race/ethnicity [*n* (%)]		69						35						34						0.36^g^
White				44 (64)						25 (71)						2 (6)				
Black				5 (7)						3 (9)						9 (26)				
Hispanic				15 (22)						6 (17)						4 (12)				
Other				5 (7)						1 (3)						19 (43)				
Occupation [*n* (%)]		70						36						34						0.000^g^
Dry-wall/taper				2 (3)						2 (6)						0 (0)				
Carpenter				22 (31)						21 (58)						1 (3)				
Painter				36 (51)						13 (36)						33 (97)				
Abbreviations: BMI, body mass index; CI, confidence interval. **a**Low BPb levels are identified as values ≤ 4 µg/dL, and high blood levels are identified as those > 4 µg/dL . **b**Except where indicated, differences between low and high Pb groups were assessed using a two-group *t*‑test with the Satterthwaite approximation. **c**The BDI-II assesses the intensity of depression in clinical and normal patients; a high score indicates a higher level of depression. **d**Individuals with higher scores have a more negative outlook (dysphoria, worry, and irritability), **e**Individuals with higher scores have more difficulties with public interactions (discomfort in social interactions, reticence, and lack of social poise.) **f**State anxiety is a component of the STAI and is a measure of the current anxiety level. **g**Determined by exact chi-square test.																				

*ACTH.* Paired *t*-tests confirmed that the TSST induced a statistically significant increase in ACTH, with concentrations 17% higher (*p* = 0.001) immediately after completion of the TSST (minute 5) and peaking at minute 15 on average. We used continuous linear regression models to evaluate the effects of BPb or TPb on ACTH. At baseline, there was a nonstatistically significant association between ACTH and BPb (*p* = 0.052), with a 19.5% increase in ACTH for an IQR increase in log-BPb. Adjusted estimates indicated that BPb and TPb were significantly associated with ACTH, with 18% and 24% higher mean levels associated with IQR increases in log-BPb (corresponding to a 2.3-fold increase in BPb) at 10 and 15 min, respectively. For IQR increases in log-TPb (corresponding to a 1.5-fold increase in TPb) we observed 9–13% increases in ACTH for the comparisons at 10, 15, and 20 min poststressor (Table 2). Although we observed similar patterns of results using the dichotomized predictive variables (median split), none of the comparisons were statistically significant except for baseline ACTH, which was higher in the higher BPb group (*p* = 0.032); mean [95% confidence intervals (CIs)] were 18.4 pg/mL (15.4–21.8) for the low BPb group and 21.5 pg/mL (17.9–25.7) for the high BPb group.

**Table 2:** Plasma ACTH concentrations (pg/mL) and adjusted BPb and TPb effects on ACTH before and after the TSST.*^a^*

Percent difference (95% CI) for an IQR increase*b*		
Time (minute)	ACTH [GM (95% CI)]	BPb	TPb
0 (baseline)		19.8 (7.5, 22.4)		19.5 (–0.1, 43.0)		5.1 (–6.3, 17.9)
5		22.7 (19.9, 25.8)		3.1 (–9.6, 17.6)		7.6 (–0.2, 16.0)
10		25.0 (21.9, 28.6)		18.0 (0.6, 38.4)		10.3 (1.6, 19.7)
15		27.8 (24.1, 32.1)		23.5 (2.1, 49.4)		13.4 (2.9, 25.0)
20		25.8 (22.7, 29.4)		15.9 (–2.0, 37.0)		9.4 (0.5, 19.1)
40		21.9 (19.4, 24.7)		9.5 (–4.9, 26.0)		6.7 (–0.6, 14.4)
60		18.9 (16.7, 21.5)		11.5 (–3.1, 28.3)		4.2 (–4.1, 13.3)
75		17.3 (14.9, 20.0)		–4.1 (–17.6, 11.6)		0.7 (–7.8, 9.9)
AUC		1,665 (1,480, 1,874)		11.5 (–2.4, 27.5)		8.6 (1.5, 16.2)
GM, geometric mean. **a**All follow-up time points are adjusted for baseline ACTH values; the model was adjusted for age, education, social inhibition, negative affect, and depression (BDI-II) scores. **b**Estimates of the percent increase or decrease in ACTH associated with an IQR increase in log-BPb or log-TPb are based on a regression model using log(ACTH) as a continuous response and log(BPb) or log(TPb) as a continuous predictor.						

*CORT.* Paired *t*-tests confirmed that the TSST induced a statistically significant increase in CORT, with concentrations 19% higher at minute 10 (*p* = 0.002) and peaking at minute 20 on average. In the continuous linear regression model adjusted for the selected covariates, CORT levels at baseline (minute 0) were significantly lower with higher BPb and TPb levels (Table 3). Specifically, CORT levels were estimated to be 19% lower in association with an IQR increase in log-BPb. After adjustment for baseline CORT and other covariates, regression analyses did not reveal significant associations of BPb or TPb with the CORT response to the stressor except in one condition; 15 min after baseline, participants with higher TPb levels had higher CORT levels (Table 3). Although not significant after adjustment for baseline, TPb appeared positively associated with CORT at other time points. Analyses with median split revealed that baseline CORT was significantly lower for participants with higher BPb levels (*p* = 0.04); mean (95% CIs) were 11.6 µg/dL (10.0–13.5) for the low BPb group and 9.8 µg/dL (8.2–11.7) for the high BPb group). No other median split comparisons were statistically significant.

**Table 3:** Serum CORT concentration (µg/dL) and adjusted BPb and TPb effects on CORT before and after the TSST.*^a^*

Percent difference (95% CI) for an IQR increase*b*		
Time (minute)	CORT [GM (95% CI)]	BPb	TPb
0 (baseline)		10.7 (9.5, 12.0)		–18.5 (–30.8, –4.0)		–10.5 (–19.3, –0.8)
5		10.8 (9.8, 12.0)		6.3 (–2.2, 15.7)		2.4 (–3.6, 8.7)
10		12.7 (11.4, 14.2)		5.2 (–8.2, 20.6)		8.4 (–0.4, 18.1)
15		14.1 (12.6, 15.8)		2.8 (–11.4, 19.3)		12.2 (2.9, 22.4)
20		14.3 (12.8, 16.0)		9.1 (–7.4, 28.5)		9.8 (–0.8, 21.5)
40		13.0 (11.6, 14.5)		–1.8 (–16.8, 15.9)		6.9 (–2.8, 17.5)
60		11.6 (10.4, 12.9)		–6.3 (–19.5, 9.1)		4.5 (–4.8, 14.7)
75		9.7 (8.7, 10.7)		–0.6 (–13.6, 14.2)		5.4 (–3.4, 15.0)
AUC		896 (813, 987)		0.7 (–10.7, 13.5)		6.2 (–1.3, 14.3)
GM, geometric mean. **a**All follow-up time points are adjusted for baseline CORT values; the model was adjusted for age, education, social inhibition, negative affect, and depression (BDI-II) scores. **b**Estimates of the percent increase or decrease in CORT associated with an IQR increase in log-BPb or log-TPb are based on a regression model using log(CORT) as a continuous response and log(BPb) or log(TPb) as a continuous predictor.						

**Figure 2:**
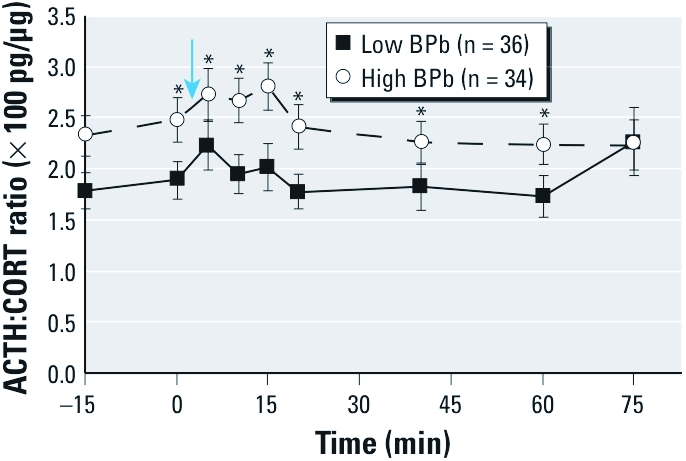
Time profile for the ACTH:CORT ratio (mean ± SE) in low (≤ 4 µg/dL) and high (> 4 µg/dL) BPb groups. The ACTH:CORT ratio illustrates the difference between these two components during the stress response in these two groups. An elevated ACTH:CORT ratio indicates a weaker adrenal responsivity to ACTH (Nater et al. 2010). The arrow indicates the execution of the TSST. *p < 0.05 for the analysis of covariance.

*ACTH:CORT ratio.* Because Pb exposure was associated with increased ACTH and lower CORT at baseline, we repeated the regression analyses using the ACTH:CORT ratio as the outcome measure (Table 4). BPb was positively associated with the ratio at all time points, although the association was not significant at 75 min. In addition, TPb was a significant predictor of the ratio at 5, 10, and 15 min poststressor (Table 4). The ACTH:CORT ratio was 14–23% greater in association with an IQR increase in log-BPb and 15–19% greater with an IQR increase in log-TPb. Figure 2 displays the average ACTH:CORT ratios over the experimental time course for the low and high BPb groups (median split). Regression analyses with the median split led to similar results (data not shown). This is indicative of a diminished adrenal responsiveness to endogenous ACTH (Nater et al. 2010).

**Table 4:** ACTH:CORT ratio and adjusted BPb and TPb effects on the ratio before and after the TSST.*^a^*

Time (minute)	ACTH:CORT ratio [GM (95% CI)]	Percent difference (95% CI) for an IQR increase*b*		
		BPb	TPb
0 (baseline)		1.9 (1.6, 2.1)		21.8 (10.3, 34.4)		17.5 (3.7, 33.1)
5		2.1 (1.8, 2.4)		14.4 (2.6, 27.5)		18.8 (3.9, 36.0)
10		2.0 (1.7, 2.3)		20.8 (9.1, 33.7)		14.7 (0.9, 30.4)
15		2.0 (1.7, 2.3)		23.1 (9.5, 38.5)		13.8 (–0.4, 30.0)
20		1.8 (1.6, 2.1)		13.8 (3.3, 25.4)		10.2 (–2.4, 24.4)
40		1.7 (1.4, 2.0)		16.9 (4.5, 30.7)		10.1 (–4.6, 27.3)
60		1.6 (1.4, 1.9)		21.6 (8.7, 36.0)		9.2 (–5.4, 26.2)
75		1.8 (1.5, 2.1)		12.3 (–0.7, 27.0)		5.7 (–8.6, 22.2)
AUC		136 (119, 155)		–12.0 (–25.8, 4.5)		–2.9 (–22.3, 21.2)
GM, geometric mean. **a**All follow-up time points are adjusted for baseline ACTH:CORT ratio values; the model is adjusted for age, education, social inhibition, negative affect, and depression (BDI-II) scores. **b**Estimates of the percent increase or decrease in ACTH:CORT ratio associated with an IQR increase in log-BPb or log-TPb are based on a regression model using log(ACTH:CORT ratio) as a continuous response and log(BPb) or log(TPb) as a continuous predictor.						

*Sensitivity analyses.* We defined “nonresponders” as individuals who failed to produce any increase above their own baseline for both ACTH and CORT responses to the TSST (i.e, values at 5 min and thereafter were equal or lower than baseline at minute 0; *n* = 5). When nonreponders were excluded, results for ACTH and CORT were similar to those reported for the entire sample (data not shown), which suggests that the apparent adrenal hyporesponsiveness to circulating ACTH was not due only to differences in the distribution of individuals who did not respond to the stressor. In addition, although SEI was not included in the final models, parallel analyses were run with and without SEI as a covariate. The direction, effect size, and significance remained similar in all analyses (data not shown).

## Discussion

In this study we estimated the effect of Pb on responses of the HPA axis to stress in individuals with BPb levels ranging from below the LOD to 31 µg/dL. Pb was significantly associated with higher ACTH:CORT ratios, which is consistent with an adrenal hyporesponsiveness to endogenous ACTH. These increases in ACTH without commensurate increases in CORT suggest a Pb-induced alteration of the HPA axis.

Psychological stress activates the HPA axis by triggering the release of CRH from the hypothalamus, which stimulates pituitary release of ACTH. ACTH then binds to the type-2 melanocortin receptor (G-protein receptor) at the level of the adrenal cortex to increase CORT release via cAMP-dependent mechanisms. Negative feedback control is provided by the circulating CORT, which binds to the glucocorticoid receptors of the parvocellular neurons to inhibit further CRH release, although negative feedback also occurs at other levels. Our data suggest that chronic Pb exposure could alter the adrenal response to ACTH, resulting in a lower CORT release than would be expected. In addition Pb could impair the negative feedback loop, leading to higher ACTH during the stress response.

Within a population, the correlation between ACTH and CORT is variable and modified by multiple parameters including age, sex, and body mass index (BMI) (Veldhuis et al. 2009). We adjusted the model for a linear effect of age, and in our sample of exclusively male participants, the inclusion of BMI as a predictive variable did not substantially change effect size estimates or the statistical significance. Interestingly, Nishiyama et al. (1985) observed that Pb reduced ACTH-induced corticosterone secretion in cultured rat adrenocortical cells. However, these authors found that Pb did not affect steroidogenesis when dibutyryl cyclic AMP was added instead of ACTH, suggesting that Pb-induced alterations occurred upstream, perhaps at the level of the melanocortin receptor. To our knowledge, none of the animal or human studies that have investigated the effect of Pb on the HPA axis have included ACTH as an outcome measure.

In the present study, CORT concentration at baseline was negatively associated with Pb. This finding is consistent with earlier reports in which plasma CORT levels were lower among Pb-exposed workers (Gustafson et al. 1989) and in Pb-intoxicated workers (Cullen et al. 1984); these studies did not, however, investigate the stress response. In contrast, Gump et al. (2008) reported that CORT reactivity following acute stress was positively associated with Pb exposure. The differences in HPA responsivity between adults and children exposed to Pb could be attributed to the different types of exposure: long-term chronic exposure versus developmental exposure, which may also affect fetal programming (Pilsner et al. 2009). In addition, the stress paradigms used by Gump et al. (2008) and in the present study were very different.

The TSST combines cognitive tasks with social evaluative threat, which has been reported to elicit the most reliable stress response (Dickerson and Kemeny 2004). In the present study, the TSST induced statistically significant increases in circulating ACTH and CORT levels peaking 15 min and 20 min, respectively, after initiation of the stress protocol; this supports the validity of the TSST as a stressor. Furthermore, the TSST is akin to real-life stressful situations involving major emotional components, whereas other stress tasks relying on experimental pain resemble physical stressors. As such, our results could be generalized to situations encountered in everyday life where social evaluation occurs, such as giving presentations before an audience or parent–teacher conferences.

Rodent studies have shown that the impact of Pb on CORT is dependent upon the period of exposure (maternal vs. lifetime), sex, and age, and, importantly, is modulated by the behavioral experience of the animals (Rossi-George et al. 2011; Virgolini et al. 2008). In rodents, maternal Pb exposure alone is sufficient to cause prolonged CORT elevations following stress challenge or dexamethasone injection, even when measured well into adulthood (Rossi-George et al. 2009). As with any cross-sectional study, it is difficult to establish the directionality of the relationships. Thus, we cannot exclude the possibility that the differences in HPA-axis function observed in our participants may alter Pb biodisposition. However it has been shown that stress exposure alone does not affect BPb levels in rodents (Cory-Slechta et al. 2004), suggesting that Pb is driving the effects.

In the present study, most of the results were more significant when comparisons were made on the basis of BPb than TPb. The most obvious reason for this difference is related to statistical power due to the larger sample size of participants with BPb measurements versus those with TPb measurements. However, we should note that although less significant, the bone lead results mirror the blood lead results.

As with all such studies, ours has limitations. These include a relatively small sample size; a limited number of individuals with high Pb exposure; and, for ethical reasons, a relatively mild and time-limited stressor. Also, sex differences in HPA-axis dysfunction following Pb exposure, which have been well documented in animals, could not be addressed in the present study because the analysis was limited to men. Moreover, the timing of Pb exposure in our participants is likely to have been variable through their lifetimes, which could interact with the documented outcomes.

Collectively, the findings presented here, as well as those previously reported in rodents and in children, suggest that Pb at levels below the reference values for workers (< 40 µg/dL) (Occupational Safety and Health Administration 1995) can alter the HPA axis at one or multiple levels. Our results suggest that Pb influenced the relationship between ACTH and CORT. Pb could modify the synchrony between ACTH and CORT by exerting a stimulatory effect on the pituitary production of ACTH, altering the negative feedback mechanism and/or inducing a hyporesponsivity of the adrenals to ACTH.

To our knowledge no animal or human studies have investigated the relation between Pb and ACTH *in vivo*. Hypothetically, Pb could alter ACTH-dependent CORT release by affecting ACTH binding to the melanocortin receptor or the downstream pathways that lead to CORT release (Doufexis et al. 2007; Halkerston 1975), but further studies are needed to really understand our findings. Future studies could evaluate in occupationally exposed workers the effect of the combined dexamethasone/CRH test (Heuser et al. 1994; Schüle et al. 2009) with serial measurements of plasma ACTH and serum CORT in association with BPb and bone Pb measurements.

## Conclusions

Our findings are consistent with a Pb-induced HPA-axis dysfunction at baseline and during stress reactivity. As such, our data add to an accumulating body of literature implicating HPA-axis dysfunction as a determinant of a variety of diseases and disorders that have also been associated with Pb exposure (Rossi-George et al. 2009).
